# Voluntary Exercise-Induced Activation of Thyroid Axis and Reduction of White Fat Depots Is Attenuated by Chronic Stress in a Sex Dimorphic Pattern in Adult Rats

**DOI:** 10.3389/fendo.2019.00418

**Published:** 2019-06-26

**Authors:** Marco Antonio Parra-Montes de Oca, Mariana Gutiérrez-Mariscal, Ma Félix Salmerón-Jiménez, Lorraine Jaimes-Hoy, Jean-Louis Charli, Patricia Joseph-Bravo

**Affiliations:** Departamento de Genética del Desarrollo y Fisiología Molecular, Instituto de Biotecnología, Universidad Nacional Autónoma de Mexico, Cuernavaca, Mexico

**Keywords:** TRH, WAT, HPT axis, restraint, social isolation, exercise

## Abstract

The activity of the hypothalamus-pituitary-thyroid (HPT) axis is inhibited by energy deficit, by acute or chronic stress, but activated by cold exposure or exercise. Because stress curtails acute cold induced activation of HPT, we evaluated the effect of chronic stress on HPT axis response to voluntary exercise, a persistent energy-demanding situation. Adult male and female Wistar rats were exposed to restraint stress, 30 min/day for 2 weeks, or to isolation (Iso) [post-natal day [PND] 30–63]. Exercise was performed (7 p.m.−7 a.m.) in a running wheel, sedentary controls stayed in individual cages (Sed); at 7 a.m. they were housed with their cage mate or individually (Iso); food intake by the exercised group was measured day and night to pair-fed Sed. At sacrifice, hormones, mRNA levels and tissue weights were quantified. Control or restrained adult rats had access to running wheel daily for 2 weeks. Compared to C, exercise decreased white adipose tissue (WAT) mass in females and males, increased hypothalamic paraventricular nucleus (PVN)-*Trh* expression in males proportionally to exercise performed, and increased TSH and T4 serum concentration in females. These changes were not detected in restrained groups. Starting at PND 63 control (2/cage) and isolated (1/cage) rats either exercised on 10 alternated nights or were sedentary. In control male animals, compared to Sed rats, exercise did not decrease WAT mass, nor changed HPT axis activity, but increased *Pomc* and deiodinase 2 (*Dio2*) expression in mediobasal hypothalamus (MBH), adrenergic receptor β3 and uncoupling protein-1 in brown adipose tissue. In control female animals, exercise decreased WAT mass, increased *Pomc, Dio2*, and *Trhde* expression in MBH, and TSH serum concentration. Iso females had lower TSH and T4 serum concentration, *Dio2* and *Trhde* expression in MBH than controls. The stress response was higher in isolated males than females, but in males it did not alter the effects of exercise, in contrast to isolated females that had a blunted response to exercise compared to controls. In conclusion, chronic stress interferes with metabolic effects produced by exercise, such as loss of WAT mass, coincident with dampening of HPT activity.

## Introduction

Physical activity and food intake are the two most important controllable variable in setting body weight. Energy expenditure is produced by basal metabolism and thermogenesis required for everyday living and physical activity. In concert with the sympathetic nervous system, thyroid hormones (TH) contribute to thermogenesis and basal metabolic rate, but they also participate in several aspects of exercise performance as they are directly involved in muscle activity, mitochondrial biogenesis, lipolysis, glycolysis, and gluconeogenesis, and mobilization of fuel to metabolically active tissues (1, 2). Patients with hypothyroidism, overt or subclinical, have reduced exercise tolerance, endurance, muscle strength, cardiovascular functions, deficient control of energy expenditure, fatigue, and poor quality of life (3–6).

Tissue levels of thyroid hormones are regulated by the activity of the hypothalamic-pituitary-thyroid (HPT) axis, and at target cells by transporters, deiodinases, and membrane or intracellular TH receptors, the latter acting as transcription factors (1, 7–9). The HPT axis is subject to multifactorial regulation, initiating with neurons of the hypothalamic paraventricular nucleus (PVN) that synthesize thyrotropin releasing hormone (TRH) and release it in the median eminence, in the vicinity of portal vessels and β2-tanycytes. These neurons receive multiple afferents from the arcuate nucleus and other brain areas that convey information of environmental, nutritional and metabolic status (10, 11). Tanycytes express deiodinase-2 (*Dio2*) that transforms T4 to T3, TH transporters, and TRH-degrading enzyme (*Trhde*) that inactivates released TRH (10). Once in portal vessels, TRH is transported to the adenohypophysis where it controls synthesis and release of thyrotropin (TSH). TSH activates synthesis of T4 in thyroid; some T4 is deiodinated to T3 in thyroid but most in target tissues (1, 12, 13). T3 is the active hormone at transcriptional level and responsible for feedback inhibition on TRH and TSH synthesis (7, 14, 15).

Multiple factors modulate the activity of the HPT axis; it is inhibited in situations of energy deficit as fasting, food restriction or chronic illness, and stimulated in certain conditions of energy excess (10, 16). Situations of energy demands as cold exposure and some types of exercise increase HPT axis activity (17–20). Rats running in a treadmill have increased serum concentration of T3 at the beginning of exercise (21) but lower after 120 min. In humans, contradictory results relate to the intensity and duration of exercise, nutritional status, and timing of blood samples; increased TSH serum concentrations are detected at short times after an exercise bout, and of T4 in some conditions although dehydration may give false results (22); increased TSH and TH concentration is detected after endurance training if measured before exhaustion but inhibited if energy reserves are temporarily depleted, or in conditions of high release of corticosterone or interleukins, for example (23). Research in animals has produced also inconsistent results; among the confounding factors, exercise usually diminishes food intake (19, 24), which by itself drives inhibition of HPT axis; likewise, for stress imposed by certain types of exercise (21). Access to a wheel provides rodents with the opportunity to exercise in voluntary non-stressed conditions (25). Using this paradigm we previously demonstrated that compared to sedentary animals exercised rats decrease their food intake and mass of white adipose tissue (WAT), and diminish the parameters that reflect HPT axis activity; however, if values are compared to a pair-fed group there is still a strong decrease in WAT mass, but PVN *Trh* expression, TSH and T3 serum concentrations are higher and proportional to the loss of WAT mass (19).

Another important regulator on HPT activity is stress, either acute or chronic, which may cause inhibition. Stress diminishes synthesis and release of TRH, TSH, and TH, and hepatic conversion of T4 into T3 (26–30); some effects are mimicked by acute or chronic corticosterone injections (31, 32). Not only basal HPT axis activity is affected by acute stress but also its response to an energy demanding situation as cold exposure; rats subjected to an acute stress or to corticosterone injection prior to cold exposure do not present the expected activation of HPT axis activity, and of a target organ as brown adipose tissue (BAT) (19, 32). The interference of glucocorticoids on neuronal activation of TRH neurons has been demonstrated at the level of *Trh* transcription in hypothalamic cells *in vitro* (33, 34); *in vivo*, corticosterone administration inhibits cold-induced CREB phosphorylation in TRH neurons (34). All these data pertain to male rodents as data on females are scarce.

Although chronic exposure to stress or to corticosterone inhibits PVN *Trh* expression and serum TSH concentration (31), the response of the HPT axis in chronically stressed animals exposed to energy demands is unknown. There are several models to induce chronic stress in rodents and depending on the type, the response of the HP-adrenal (HPA) axis (CRH:ACTH:glucocorticoids) can either subside (animals habituate) or intensify. Chronic stress induced by daily intermittent short periods of restraint (Res) is a model of psychological stress that produces habituation of the response of the HPA axis, but if rodents are submitted to a new stressor, response is enhanced (35, 36). After 30 min of acute restraint, PVN *Trh* expression and TSH serum concentration decrease but levels normalize after 14 daily sessions (19, 37). The cold-induced activation of the HPT axis is also curtailed in male rats submitted to 14 days of intermittent repeated restraint (unpublished). Another type of stressor is individual housing; isolation (Iso) constitutes a continuous chronic stress recognized as a strong psychosocial stressor that causes hyperactivation of the HPA axis, and the lack of social stimuli prevent habituation (38); animals isolated since puberty have behavioral and physiological malfunctions, as puberty and adolescence are critical developmental periods for circuit formation between limbic areas (39, 40).

As an adequate response of the HPT axis to exercise favors mobilization of energy reserves (19, 41), we hypothesized that chronic stress attenuates the response of the HPT axis to voluntary exercise and this contributes to long-term consequences in WAT depots. We selected these two paradigms to distinguish the effect of stress on animals that habituated (Res) and no longer have high basal corticosterone levels but may show an exaggerated response to a novel situation as exercise (35–37), compared to isolated animals that do not habituate and show disfunction of HPA axis (42). As an acute corticosterone increase blunts the increase in TRH synthesis induced by cold-induced neuronal stimulation (32–34), we may expect a different response if effects depend only in corticosterone variations or, if they depend on limbic circuitry (43). We studied the response of the HPT and HPA axes (the latter as control) and WAT depots to voluntary wheel running in male and female rats submitted to two types of chronic stress: (a) 2 weeks of intermittent restraint in adult animals, and (b) isolation caused by keeping rats in individual housing since puberty.

## Materials and Methods

### Animals

Male and female Wistar rats were raised at the Institute's Animal Facility, as described in Sotelo-Rivera et al. (32). Breeding colony management and care was carried out only by two technicians to avoid additional stress. The breeding procedure followed: an outbreed monogamous pair scheme in which the male is removed before the litter is born; litter is adjusted to 10 offspring at post-natal day 2 (PND 2) and culled if necessary. Animals are weaned at PND 21, placed by sex in groups of 2–4/cage and treated as described (44). Appropriate records assure non-inbreeding; colony breeders are renewed every 5 years. For these experiments, rats (PND 45–60) were placed in experimental independent rooms according to sex (2/cage). When males were near 350 g or 2.5 months, they were housed in groups of two per cage, in cages with a wire bar lid modified so that the rear 8.00″ of lid is ~3.00″ higher (total height of 11.00″), allowing rats to stand on their back legs (Y Corporation of America, INC.). Food (Teklad 2018SX, Envigo, USA) and water were offered *ad libitum* except where indicated otherwise. Maintenance and work with animals followed the Guide for the care and use of laboratory animals (8th ed.), as well as the Mexican norm NOM-062-ZOO-1999. These experiments were approved by the Bioethics Committee of the Institute, approval No. 273 and 318.

### Experiment 1. Chronic Intermittent Restraint

Forty adult male rats at PND 86 (390 ± 6 g) were weighed and separated in five groups (eight rats per group). Two groups were taken to an empty room and introduced into a plexiglass tube (23.5 × 7 cm) with slots for ventilation and keeping tail out, in prone position during 30 min every day at 10:00 h for 2 weeks [restrained [Res] group]; two groups serving as experimental controls (C) were taken to a different room and placed in a small cage during 30 min (37). A group of 8 naïve (N) animals from the same cohort was kept undisturbed in the animal room to evaluate environmental conditions effect. Food intake was quantified by weight difference: A set measured amount of food was placed over the rack of the Res and N groups every 3 days, the amount left was weighted again after recovering small pieces from the bed. The mean of Res intake offered to controls (pair-fed).

At PND 101, rats of C and Res groups were divided; half of C (420 ± 13 g) and of Res (429 ± 14 g) animals were introduced every night (7:00 p.m.−7:00 a.m.), during 2 weeks, in a cage with a running wheel of 25 cm diameter (AccuScan Instruments INC) for voluntary exercise (exercised: Ex), the other two halves of animals stayed in individual cages (sedentary: Sed) (19). All rats rejoined their cage mate in the morning. Food was offered during dark or light cycles and intake registered at every food change (between 7:00 a.m. and 9:00 a.m. in the morning and 5:00 p.m.−7:00 p.m. in the night). Sedentary rats were pair-fed to the intake of Ex rats.

The same paradigm was used to study female rats starting at PND 95 (before Res: 248 ± 4 g; after Res, before Ex: 253 ± 3 g).

### Experiment 2. Social Isolation

At PND 30 male (*N* = 32; 110 ± 2 g) and female (*N* = 32; 100 ± 2 g) Wistar rats were separated in two groups that were housed, either in individual cages (Iso) (*N* = 16) or in groups of 2/cage (C) in the same room to expose isolated rats to visual, auditive, and olfactory cues of other rats (45). Rats were maintained as described (32), in separate rooms according to sex. Food and water were offered *ad libitum*, food intake (FI) was quantified every 3 days from PND 30 to PND 63 and body weight measured every week in the adolescence period. At PND 63 C (Males: 379 ± 8 g; Females: 223 ± 6 g) or Iso (Males: 363 ± 7; Females: 224 ± 5 g) rats of each sex were further separated in two groups, one was left undisturbed (Sed) and the other exposed to a running wheel (Ex) (*N* = 8 each) as described above and in Uribe et al. (19), with some modifications. Due to limited amount of running cages, rats had alternate access during 16 days for a total of 10 running days. At light change (7:00 a.m.), C animals were housed with their cage-mate or in an isolated cage (Iso group); food intake, and number of revolutions were recorded daily and body weight every 2 days. Sedentary rats received the amount of food consumed by the exercised group as described previously.

### Sacrifice and Tissue Collection

On the last day of exercise, 3 h after lights on and cage change, animals were decapitated with a sharp guillotine by an experienced technician as described (19), in a separate laboratory near the animal rooms, taking one animal at a time, cleaning the guillotine in-between and with care of diminishing stress. Trunk blood was collected, serum separated and stored in aliquots. Adipose tissues (gonadal, retroperitoneal and interscapular) and adrenals were extracted and weighed in fresh; brain and BAT were placed in dry ice and stored at −70°C. Several experienced persons participated to limit time between initiation and termination of sacrifice to a maximum of 2 h. Dissection of hypothalamic regions is described in Supplementary Figure 1.

### Hormone Assays

Radioimmunoassays were utilized for TSH (NIDDK reagents and protocols) and corticosterone (CORT; reagents from Merck-Millipore, PerkinElmer, and Sigma) quantification. Total T3 and T4 were quantified with ELISA kits (Diagnóstica International Zapopan, JAL, México) adding to standard curves an aliquot of hypothyroid rat serum (46). All samples were measured in duplicate and the mean taken as one determination; intraassay and interassay coefficients of variation were <10%.

### Semi-quantification of mRNA

Total RNA was extracted from frozen tissues according to Chomzynsky et al. (47) with some modifications for BAT, for which a centrifugation (870 g, 4°C, and 10 min) and an extra chloroform wash were performed to remove fat. Relative mRNA levels were measured by RT-PCR according to reported conditions (19); for those not previously reported by our group, linearity was verified and optimized conditions described in Supplementary Figure 2.

### Statistical Analysis

The mean of duplicate biochemical measurements was considered as one determination; results were calculated as percent of each experiment's control group mean. Two-way ANOVA was performed to analyze the effect of chronic stress and sex before exercise, and to analyze the effect of chronic stress and physical activity on the different variables. Three-way ANOVA was performed to analyze effects of chronic stress, physical activity and sex, and interaction between these variables. All data were analyzed by Sigma plot 11.0 (Systat Software, Inc.) and GraphPad Prism 8 (GraphPad Software, Inc.). Results of statistical analysis are described in Supplementary Tables 1, 2. *Post-hoc* comparisons were carried out with either Bonferroni or Holm-Sidak tests, and difference considered to be significant when *P* < 0.05.

## Results

### Experiment 1. Effect of Chronic Restraint Stress on the Response to Voluntary Exercise

#### Chronic Restraint Decreased Food Intake and Body Weight Gain

During the period of intermittent restraint, there was no difference in the total food intake or body weight gain (BWg) of restrained male rats compared to naïve (Table 1). Res females ate 10% less than the naïve group and as controls were pair fed to Res, they ate all that was offered (Table 1). BWg was lowest in the pair-fed, followed by Res and highest by N. Restraint diminished voluntary food intake, pair-fed were probably stressed by hunger causing highest weight lost. Females ate less absolute food than males (Table 1) but ate more relatively to body weight, thus their food efficiency was lower (Supplementary Table 3A).

**Table 1:** Food intake (FI) and body weight gain (BWg) during restraint stress period of naive (N) pair fed controls (C) and restraint (R) male and female rats.

	**N**	**C**	**Res**
**MALES**			
FI (g/day)	26.6 ± 9.4	27.2 ± 0.6	26.4 ± 0.8
BWg (g)	31.6 ± 3.5	27.4 ± 3.5	25.3 ± 2.4
**FEMALES**			
FI (g/day)	20.7 ± 0.11^A^	18.41 ± 0.26°^A^	18.1 ± 0.2°^A^
BWg (g)	10.25 ± 3.9^A^	−4 ± 2.12°^A^	5.5 ± 1.5^*^^A^

Male and female Wistar adult rats were restrained (R) or kept in single cage for 30 min/day for 14 consecutive days in two independent rooms, food intake of restrained group was pair-fed to control group (C). Results are expressed in mean ± SEM. Significant ANOVAs (Supplementary Table 1A) followed by post-hoc:

* P < 0.05 vs. C group;

A P < 0.05 vs. Sex;

° *P < 0.05 vs. N*.

#### Chronic Restraint Diminished Motivation for Running Only in Males

**Figure 1:**
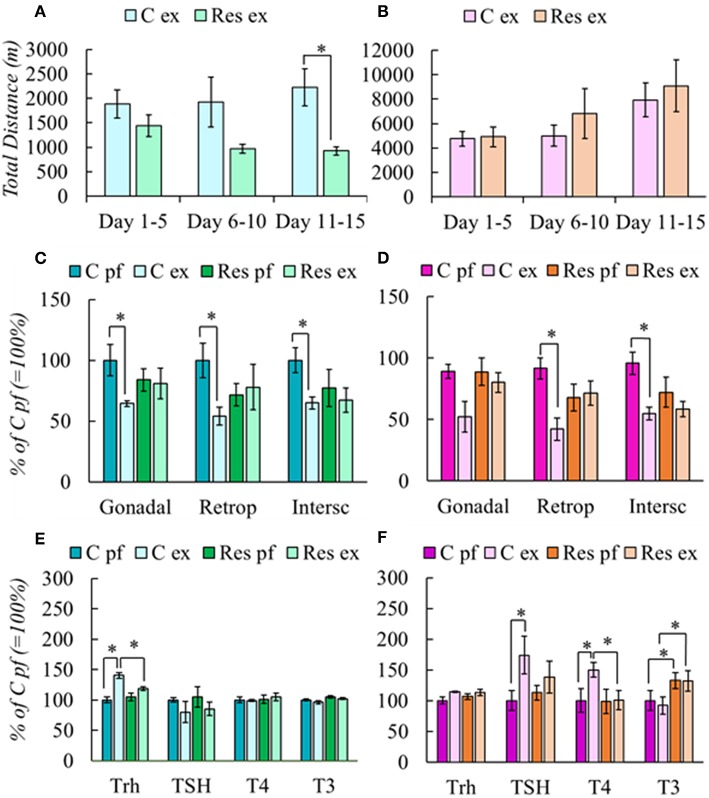
Effect of chronic restraint on physical activity **(A,B)**, fat mass **(C,D)**, and HPT axis response **(E,F)** of male and female rats. Stress protocol and exercise conditions as described in Tables 1, 2. respectively. Physical activity was expressed in total distance in meters at periods stated in the abscissa. Results are expressed in percent of mean values of C pf. Significant ANOVAs (Supplementary Table 2A), followed by *post-hoc*: ^*^P < *0.05*.

Res males performed less exercise than controls (Figure 1A) whereas females were not affected as both groups gradually increased the total distance ran which were considerably higher than those ran by males (Figure 1B). As previously published (19), exercise diminished food intake more in C-Ex than in Res-Ex; body weight gain in Sed or Ex, C or Res was 5% lower than N but differences between C or Res groups were non-significant (Table 2). Compared to N, exercise decreased food intake in C or Res females but BWg was not significantly different (Table 2 and Supplementary Table 4A).

**Table 2:** Food intake (FI), Body weight gain (BWg) and distance per day (DPD) after exercise period of male and female rats.

	**N**	**C pf**	**C ex**	**Res pf**	**Res ex**
**MALES**					
FI (g/day)	25.15 ± 0.97	18.5 ± 0.9°	17.8 ± 1.1°	21.5 ± 1.9°	20.9 ± 1.2°
BWg (g)	19.8 ± 10.2	−6.5 ± 3.6°	−12.75 ± 2.9°	2.4 ± 4.7°	−9.2 ± 13.4°
DPD (m)	–	–	402 ± 78	–	280 ± 64
**FEMALES**					
FI (g/day)	23.27 ± 0.54	15.39 ± 1.58°	15.55 ± 1.52°	16.12 ± 1.35°^A^	16.28 ± 1.35°^A^
BWg (g)	15.3 ± 5.46	5.0 ± 4.0^A^	7.6 ± 2.2^A^	15.6 ± 3.8^A^	15.8 ± 2.8^A^
DPD (m)	–	–	1179 ± 186^A^	–	1599 ± 248^A^

Effect of restraint.

Restrained or control rats were either exposed to a running wheel (C ex and Res ex) or left individually in a cage (C pf and Res pf) overnight for 14 days. Food intake of exercised animals was pair-fed to control groups. Significant ANOVAs (Supplementary Table 2A) followed by post-hoc:

A P < 0.05 vs. Sex;

° *P < 0.05 vs. N*.

#### Chronic Restraint Blunted Exercise-Induced Loss of Fat Mass and Activation of HPT Axis in a Sex-Dependent Manner

Exercise induces WAT mass loss even if it is not evident in body weight (19, 48). C males reduced gonadal, retroperitoneal, and interscapular fat depots after exercise, but Res group did not (Figure 1C). C-Ex females also reduced them, although gonadal WAT loss was not significant; as in males, Res females did not lose WAT after exercise (Figure 1D).

Because chronic stress affects the HPA response to new stressors and exercise has been considered either to increase or diminish HPA activity (19, 49), we measured gene expression profile of *Crh* and *Gr* in PVN, and corticosterone in serum. There were no significant differences in any of these variables in groups of either sex (not shown), except for an increase in right-adrenal weight of Res males (N, 27 ±3; C-Ex 31 ± 1; Res 31 ± 3; Res-Ex 39 ± 2^*x^ mg, ^*^P = *0.007* vs. N; ^x^*P* = *0.02* vs. C-Ex) suggesting chronic stress (50). Females had higher basal corticosterone serum concentration (181 ± 7 ng/ml) than males (65 ± 4 ng/ml); but in contrast, values were increased similarly in all experimental groups compared to naïve (C-Sed 396 ± 56, C-ex 332 ± 19, Res-Sed 354 ± 30, Res-Ex 303 ± 16).

*Trh* expression was stimulated in C-Ex (Figure 1E) and values correlated negatively with WAT retroperitoneal mass (*r* = −0.832; *P* = *0.003*). No other parameters of HPT axis varied among male groups. In females, *Trh* expression was not significantly different between C-Ex and C-Sed or between Res groups. In contrast to males, TSH serum concentration increased in C-Ex females as well as T4 (Figure 1F). In both sexes, activation of the HPT axis coincides with WAT mass decrease only in controls, but not in those previously stressed by daily restraint.

### Experiment 2. Effect of Individual Housing on the Response to Voluntary Exercise

#### Social Isolation Did Not Affect Food Intake and Body Weight Gain During Adolescence

We chose to analyze social isolation since adolescence because this stressor prevents proper development of brain, causing behavioral and physiological alterations that can remain until adult stage (43, 51, 52). Social isolation in adulthood delays exercised-induced hippocampal neurogenesis in male and female rats, affecting more in males than in females (47, 53).

Rats were isolated at PND 30, as expected males grew faster than females; no difference was observed in body weight gain between C or Iso groups; by PND 60, body weight of C or Iso groups were similar so as their food intake (Table 3 and Supplementary Table 3B). The lack of effect of social isolation since weaning on ponderal variables during the growing phase has been previously reported (54, 55).

**Table 3:** Food intake (FI) and body weight gain (BWg) during adolescence-adulthood period of group-housed (C) and isolated (Iso) male and female rats.

	**C**	**Iso**
**MALES**		
FI (g/day)	27.58 ± 0.37	27.14 ± 0.64
BWg (g)	232.63 ± 5.48	220.00 ± 4.34
**FEMALES**		
FI (g/day)	19.55 ± 0.23^A^	19.81 ± 0.53^A^
BWg (g)	104.94 ± 3.57^A^	104.19 ± 3.06^A^

Male and female Wistar rats were kept isolated (Iso) or in 2/cage (Controls, C) from PND 30 to PND 63. Results are expressed in mean ± SEM. Significant ANOVAs (Supplementary Table 1B) followed by post-hoc:

A *P < 0.001 vs. Sex*.

#### Ponderal Changes and HPA Responses to Exercise Were Affected by Social Isolation Dependent on Sex

Exercise reduced their food intake 19% in C and 18% in Iso groups compared to average intake before exercise in both sexes (Tables 3, 4), but Iso-Ex ate more than exercised controls (Table 4), sedentary group were pair-fed accordingly.

**Table 4:** Food intake (FI), body weight gain (BWg) and distance per day (DPD) after exercise period of group-housed (C) and isolated (Iso) male and female rats.

	**C-Sed**	**C-Ex**	**Iso-Sed**	**Iso-Ex**
**MALES**				
FI (g/day)	24.74 ± 0.02	25.24 ± 0.84	26.05 ± 0.04^*^	26.30 ± 0.92^*^
BWg (g)	16.67 ± 1.20	15.00 ± 3.84	30.83 ± 2.27^*^	25.50 ± 2.53^*^^&^
DPD (m)	–	542 ± 36	–	570 ± 74
**FEMALES**				
FI (g/day)	16.99 ± 0.01^A^	17.07 ± 0.34^A^	18.16 ± 0.02^*^^A^	18.00 ± 0.46^*^^A^
BWg (g)	10.86 ± 2.98^A^	7.17 ± 3.63^A^	14.00 ± 1.41^*^^A^	13.14 ± 1.98^*^^A^
DPD (m)	–	3,l061 ± 597^A^	–	3,753 ± 522^A^

At PND 63, group-housed (C) and isolated (Iso) rats were separated in two groups, one was left undisturbed (Sedentary, Sed) and the other was exposed to a running wheel in alternated days during the dark period. Sed group received the amount of food that Ex group. Results are expressed in mean ± SEM. Significant ANOVAs (Supplementary Table 2B) followed by post-hoc:

* P < 0.05 vs. C group;

& P < 0.05 vs. Sed group;

A *P < 0.05 vs. Sex*.

**Figure 2:**
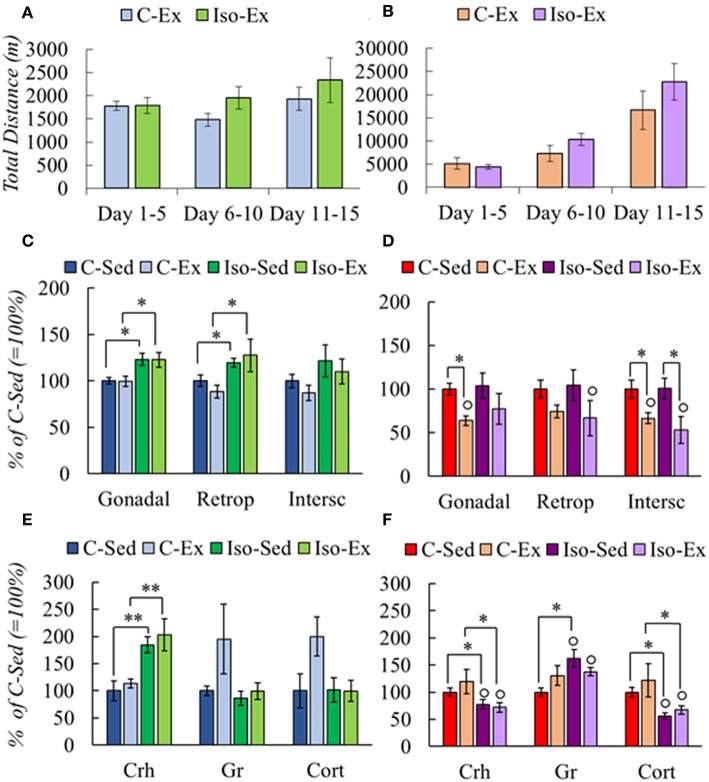
Effect of social isolation on physical activity **(A,B)**, fat mass **(C,D)**, and HPA axis response **(E,F)** of male and female rats. Rearing and exercise conditions described in tables 3 and 4, respectively. Significant ANOVAs (Supplementary Table 2B) followed by *post-hoc*: ^*^P < *0.05*; ^**^P < *0.001*; °*P* < *0.05* vs. Males of the same group.

After exercise, Iso gained more weight than controls (Table 4). If food intake was calculated in relation to body weight, females ate more and their food efficiency was lower than males (Supplementary Table 4B). Isolation did not diminish the amount of running in males or females compared to group-housed controls (Figures 2A,B), and as in the previous experiments, females ran more than males (Table 4). Total distance ran by C males in this experiment was similar to controls of experiment 1, whereas females ran more, even though they had access to the wheel only in alternate days for a total of 10 nights, instead of 14 daily nights in the previous experiment (Supplementary Figure 3); probably be due to the lighter weight of the latter (56), and/or the form of exercise, on alternate days in Iso that avoided habituation (25) compared to every day in Res.

C males had less gonadal and retroperitoneal WAT mass compared with Iso group, but there was no lost of any fat type by exercised (Figure 2C). C-Ex females lost gonadal and subcutaneous WAT mass albeit only subcutaneous in the Iso-Ex group (Figure 2D).

HPA response differed between groups. Isolation increased PVN *Crh* expression whether or not they exercised; there was a tendency to increase PVN *Gr* expression and Cort serum concentration in C-Ex but did not achieve significance (Figure 2E). Females in contrast, had lower PVN *Crh* expression and serum Cort concentration in Iso than C groups. PVN *Gr* mRNA levels increased although significant only in Iso-Sed (Figure 2F). Higher expression of *Gr* may contribute to overcome the reduced levels of corticosterone to lower *Crh* expression (57).

#### Social Isolation Altered the HPT Response to Voluntary Exercise in a Sex-Dependent Manner

**Figure 3:**
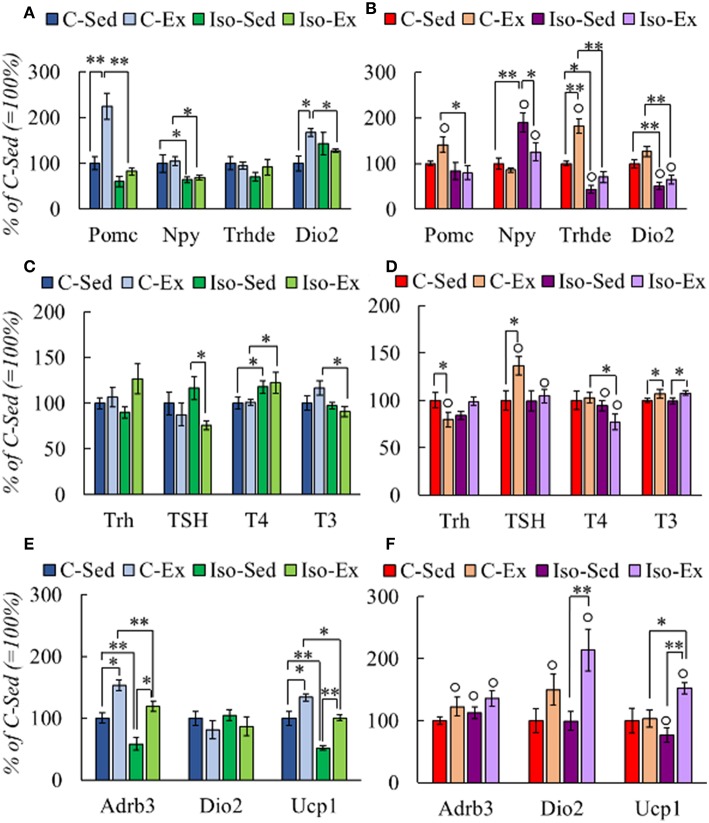
Effect of social isolation and alternate exercise on the expression of HPT status-related genes **(A,B)**, HPT axis response **(C,D)**, and genes that response to thyroid hormones in BAT **(E,F)** of male and female rats. Rearing and exercise conditions described in tables 3 and 4, respectively. Results are expressed in percent of mean values of C-Sed. Significant ANOVAs (Supplementary Table 2B) followed by *post-hoc*: ^*^P < *0.05*; ^**^P < *0.001*; °*P* < *0.05* vs. Males of the same group.

Two important regions that regulate HPT axis activity reside in mediobasal hypothalamus (MBH), the arcuate nucleus and the median eminence. In the arcuate nucleus, neuronal populations that express anorexigenic (POMC/CART) and orexigenic (NPY/AgRP) peptides stimulate or inhibit *Trh* expression in PVN, respectively (10). Tanycytes in median eminence inhibit TRH expression through deiodinase 2 activity increasing T3 levels in the hypothalamus, and the activity of TRH-degrading enzyme controls TSH secretion (58). In the MBH, the expression of arcuate peptides varied due to rearing condition or exercise. *Pomc* expression increased in C-Ex males compared with C-Sed and Iso-Ex, and *Npy* expression decreased in Iso (Figure 3A). The pattern of *Pomc* expression was similar in female MBH, however *Npy* mRNA levels were higher in Iso than C groups (Figure 3B). *Dio2* expression was higher in C-Ex than C-sed in males but *Trhde* was not modified (Figure 3A). In females, *Dio2* was higher in C than in Iso; *Trhde* increased by exercise in C and slightly in Iso (Figure 3B).

HPT axis was not stimulated by exercise in C-males. In contrast, isolated males had diminished TSH serum concentration after exercise; that of T4 augmented but in both Iso groups compared to their controls (Figure 3C). Negative correlation was observed between T4 and WAT retroperitoneal mass in C-Ex group (*r* = −0.856; *P* = *0.030*) whereas positive, in Iso-Ex (*r* = 0.879; *P* = *0.009*). In females, *Trh* mRNA levels were lower in C-Ex than in C-Sed, TSH serum concentration increased by exercise in C, and T4 decreased compared to Iso-sed, while T3 concentration increase in both exercised groups (Figure 3D). T4:T3 ratio was higher in Iso than C, and was higher in females than males (Supplementary Table 5).

Brown adipose tissue (BAT) is a target of thyroid hormones that in concert with the adrenergic system is responsible for thermogenesis, activity that contributes to energy expenditure (19, 59). Noradrenergic signaling stimulates *Dio2* expression, rising T3 concentration in the tissue (60) and, in turn, stimulates the transcription of β-adrenergic receptors (β-AR) and UCP-1 (2, 61). We thus measured the expression of these molecules; mRNA levels of *Adrb3* or *Ucp1* were lower in Iso groups of males than C but increased in both by exercise, those of *Dio2* were not changed (Figure 3E). Females showed no changes in *Adrb3* expression, decreased that of *Ucp1* in Iso-Sed compared to C-Sed while increased, as *Dio2*, in Iso-Ex (Figure 3F).

## Discussion

Life of laboratory rodents, in controlled environments, is very distant from that in natural conditions. Experimental rats have little stimuli and space for physical activity, and usually become obese (62). Although distinct from training exercise, voluntary running for short bouts provides many benefits for animals' health (63), and allows comparing the physiology of a sedentary animal with an active one. In just 2 weeks of daily running during their active phase, male rats lose WAT mass proportionally to their physical activity and to the levels of serum thyroid hormones or PVN *Trh* expression (19). Stress inhibits the response of the HPT axis to the short-term energy needs of an acute exposure to cold [(32, 34) and unpublished], but its effect on long-term energy demands was not known. We considered voluntary running a suitable model of energy demand, to study the effects of two types of chronic stress on the responses of the HPT axis to exercise in both male and female rats, given the sex dimorphism in metabolic and stress responses. A short time of exercise was selected to avoid allostatic adjustments that could make it difficult to validate results with previously reported results (19).

The chronic stressors chosen were intermittent restraint at adulthood, and isolation from puberty to adulthood. Restraint is a well-studied model of psychological homotypic stress that causes habituation (35) as well as neurological changes that cause a hyper-reactive response to a new stressor, persistence of oxidative stress, modifications in immune responses, to name a few (64). In contrast, social isolation is a model of psychosocial stress that alters glucocorticoid feedback through changes in neurochemical functions of limbic and cortical systems, affecting the maturation of some of these circuits leading to long lasting alterations that induce depression and anxiety in adult rodents (38, 65, 66); isolation during adolescence produces HPA axis malfunction at adult stage, decreasing basal levels of serum corticosterone and producing a hyper-reactive response to acute stress (67–69). Voluntary exercise attenuates various deleterious effects produced by stress, and although it may cause increases in serum corticosterone concentration it is not considered a strong stressor, in contrast to forced training or exhaustive exercise (70, 71). The exercise paradigm utilized was not considered as a new stressor since variables of HPA axis were not different in exercised to those of sedentary rats. In our experiments, both types of stress affected feeding and exercise task; Res males exercised less and ate more, whereas Iso animals, especially females, increased food intake and body weight gain, suggesting stress-induced imbalances in eating behavior. Decreased fat mass was expected in exercised rats (19, 72, 73), and was detected in adult males and females that exercised every night (experiment 1). However, animals that exercised alternately (experiment 2) did not reduce food intake as did those running every night (this experiment, and 19). In males, lipolysis induced by daily exercise appeared to reduce hunger; signals required for this and for loosing WAT mass might not be enough if exercise is not done daily. Conversely, female controls of both experiments did loose WAT mass in response to voluntary exercise but also ran considerably more than males, as is generally recognized in rodents (74, 75). They lost subcutaneous WAT (interscapular) and only one of the abdominal types (gonadal or retroperitoneal) of WAT depending on the experiment. Previous Res-stress reduced the amount of exercise performed only in males that showed no WAT mass loss; less exercise may account for the lack of fat mass lost however, stressed females that ran the same amount as controls did not lose WAT mass either, despite not differing in food intake. In isolated females, exercise induced similar tendencies in WAT mass reduction, which was significant only in subcutaneous type. These results point for a negative effect of chronic stress on the adequate WAT-metabolic response to exercise.

Voluntary and forced exercise increase basal corticosterone serum concentration (47, 69, 76) without affecting the expression of *Crh* and *Gr* in PVN (77, 78). Adult control animals of both experiments showed no exercise-induced variations in any of the HPA axis parameters, and neither those that had been previously restrained. Isolation induced HPA malfunction differently in males and females; males had increased PVN *Crh* expression but no changes in serum corticosterone concentration, whereas females had decreased serum corticosterone concentration and *Crh* mRNA levels, suggesting hypoactivity of the axis (79). These findings agree with a higher susceptibility of females to stress (80), isolation (81) and depression (82, 83), the latter usually coincident with hypoactivity of HPA (79); this might be due to the effect of stress at adolescent stage (84), and exercise could reverse the deleterious effects of stress (53, 71).

Exercise promotes the expression of *Pomc* in arcuate nucleus (ARC) (85), in agreement with what we found in C male and female rats, albeit it was not according to the amount of exercise performed, as Iso males ran similar distances and their response was blunted. Isolation and not wheel running increased ARC *Npy* expression in females, coincident with higher food intake and some reports (86, 87).

**Figure 4:**
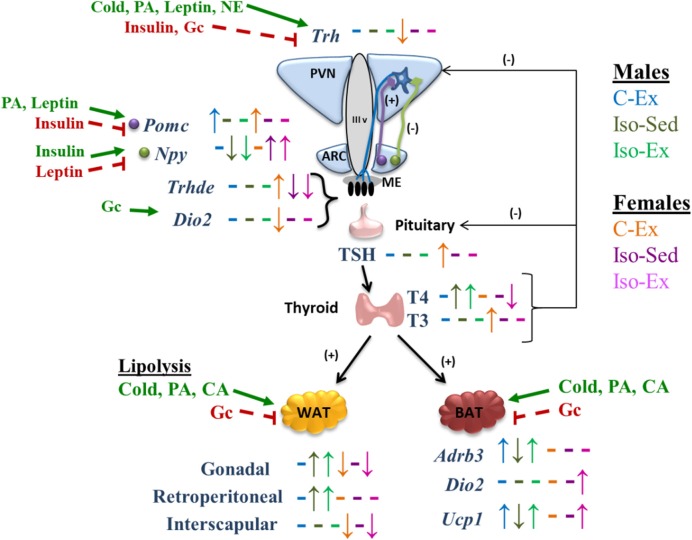
Schematic representation of the effect of social isolation and alternate voluntary exercise on HPT axis response, its regulation, and its effects in WAT and BAT. PA, physical activity; NE, norepinephrine; Gc, glucocorticoids; CA, catecholamines. Up and down arrows indicate changes and dashes indicate not changes of each experimental group compared with C-Sed group of each sex. Bold green arrows indicate activation and dashed red lines indicate inhibition.

The HPT axis is subject to multiple metabolic, hormonal and neuronal effectors that regulate its activity at multiple levels (8, 11, 56). Elements of the HPT axis showed a sex-dimorphic regulation (Figure 4). Change-dynamics of HPT axis parameters depend on the type of stimulus; PVN *Trh* expression changes can be rapid and transient (17, 18), half-lives of serum TSH and T3 are short (min to h) compared to that of serum T4 (8). Furthermore, since the activity of tissue deiodinases is also regulated, circulating thyroid hormones (TH) concentrations are difficult to interpret (12), making it necessary to evaluate as many variables as possible, if only one time point is studied. Exercise increased *Dio2* expression in median eminence (ME) in C males, possibly because of increased serum corticosterone concentration (88). If DIO2 activity was indeed increased, it should have augmented local T3 concentration which in turn, would be expected to stimulate ME *Trhde* expression (58) or inhibit PVN *Trh* expression (10), albeit with unknown dynamics. None of these changes were found in males; since animals were sacrificed 3 h after switching to a wheel-free cage, and time since the last exercise boot was not recorded, it is possible that the time-point was inadequate. However, exercise did increase ME *Trhde* expression in C females, with only a slight non-significant tendency for *Dio2* expression, and diminished PVN *Trh* expression. In females, isolation decreased ME *Dio2* and *Trhde* expression, coincident only with decreased corticosterone serum concentration, which stimulates *Dio2* expression (88); parallel changes in *Dio2* and *Trhde* reflect the dependence of DIO2 activity to set the levels of T3 in tanycytes, which activate *Trhde* expression (58). However, despite increased *Trhde* expression in ME, TSH serum concentration was increased, most probably by an exercise-induced effect that could either increase TRH release in spite of not finding a higher expression or, increased TSH release due to higher expression of TRH-R1 or in response to events distinct from TRH.

Thyroid hormones regulate the activity of adipose tissue genes which constitute additional markers of HPT axis activity. BAT can promote energy expenditure through facultative thermogenesis induced by sympathetic nervous system activity (89). The impact of exercise on BAT is still not well-understood (90) since *Adrb3* and *Ucp1* regulation in BAT by exercise depends on species and training time; in mice, treadmill training increases *Ucp1* expression but *Adrb3* expression does not change (91), while in rat *Ucp1* and *Adbr3* mRNA levels do not change after one session of treadmill exercise (92), in contrast to an increase during progressive treadmill (93). The increase in the expression of *Adrb3* and *Ucp1* in Iso-Ex males, and of *Dio2* and *Ucp1* in Iso-Ex females, suggests that voluntary exercise promotes the effect of thyroid hormones on BAT, promoting energy expenditure as heat instead of muscle work, and being able to revert the effects of social isolation.

To conclude, present results demonstrate that a history of chronic stress curtails the adequate metabolic response to voluntary exercise characterized by loss of WAT depots, in male and female rats. Females ran much more than males, which allowed them to lose fat independent of schedule (daily or alternate days). Restraint stress reduced exercise in males, either due to fatigue or lack of motivation. Some changes in the HPT axis (PVN *Trh* expression or serum concentrations of TSH or TH) were related to WAT loss, but only in controls and not in stressed groups. We propose that in control animals the HPT axis is activated transiently at each exercise boot contributing to lipolysis, event inhibited by previous chronic stress, leading to problems with fuel availability.

## Data Availability

The raw data generated are available on request to corresponding author.

## Ethics Statement

The experiments were approved by the Bioethics Committee of the Institute, approval No. 273 and 318.

## Author Contributions

MP-M, MG-M, and MS-J performed the experiments. MP-M, LJ-H, and PJ-B analyzed the data. MP-M and PJ-B wrote the manuscript draft. LJ-H and J-LC helped with manuscript edition. MP-M is a doctorate student and main contributor. PJ-B is principal investigator of grant and project director. J-LC and PJ-B are co-directors of the Neuroendocrinology group.

### Conflict of Interest Statement

The authors declare that the research was conducted in the absence of any commercial or financial relationships that could be construed as a potential conflict of interest.
