# Supplementary material for: Voluntary Exercise-Induced Activation of Thyroid Axis and Reduction of White Fat Depots Is Attenuated by Chronic Stress in a Sex Dimorphic Pattern in Adult Rats
Source: Front Endocrinol (Lausanne). 2019 Jun 26;10:418. doi: 10.3389/fendo.2019.00418 (PMC6607407; doi:10.3389/fendo.2019.00418)
Supplement: Supplementary file 1 [file Image_1.pdf]

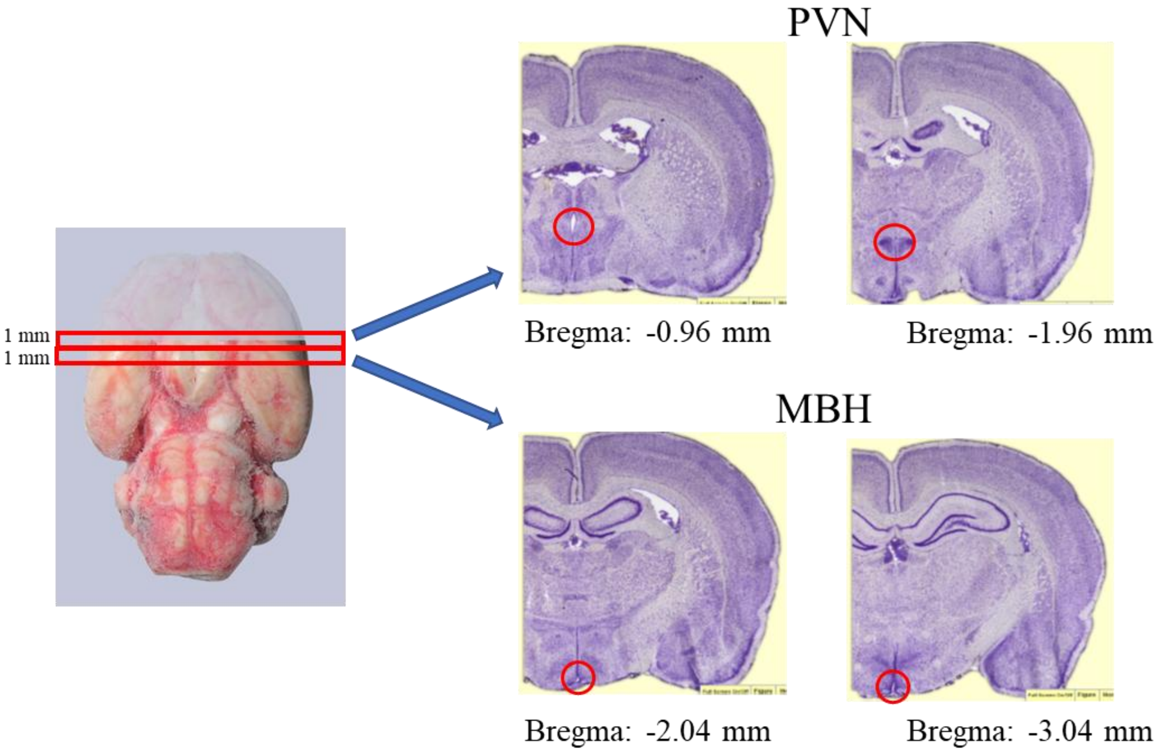

Supplementary Figure 1. Hypothalamic regions were obtained from frozen coronal slices. First slice (Bregma: -0.96 to -1.96 mm; Paxinos and Watson, 2005) included the hypothalamic paraventricular nucleus (PVN), this was dissected using a sample corer with an internal diameter of 1 mm (Fine Science Tools). Second slice (Bregma: -2.04 to -3.04 mm; Paxinos and Watson, 2005) included the medial basal hypothalamus area (MBH) conformed by arcuate nucleus and median eminence, this was dissected using a sample corer with an internal diameter of 0.5 mm (Fine Science Tools).
