# Supplementary material for: Voluntary Exercise-Induced Activation of Thyroid Axis and Reduction of White Fat Depots Is Attenuated by Chronic Stress in a Sex Dimorphic Pattern in Adult Rats
Source: Front Endocrinol (Lausanne). 2019 Jun 26;10:418. doi: 10.3389/fendo.2019.00418 (PMC6607407; doi:10.3389/fendo.2019.00418)
Supplement: Supplementary file 2 [file Image_2.pdf]

Gene	Reference/ID	Size	Sense	Antisense	Tm (°C)	Cycles	cDna (µl)
Pomc	Leriche et al., 2007	678	GAGATTCTGCTACAGTCGCTC	TTGATGATGGCGTTCTTGAA	64	26	4
Npy	de Rijke et al., 2005	220	TATCCCTGCTCGTGTGTTTG	GTTCTGGGGGCATTTTCTG	64	26	4
Adrb3	NM_013108.2	351	TCTGTGCTGGCTGCCCTTCTT	CTTCTCCTCCCCCAACCCTCAA	64	27	4

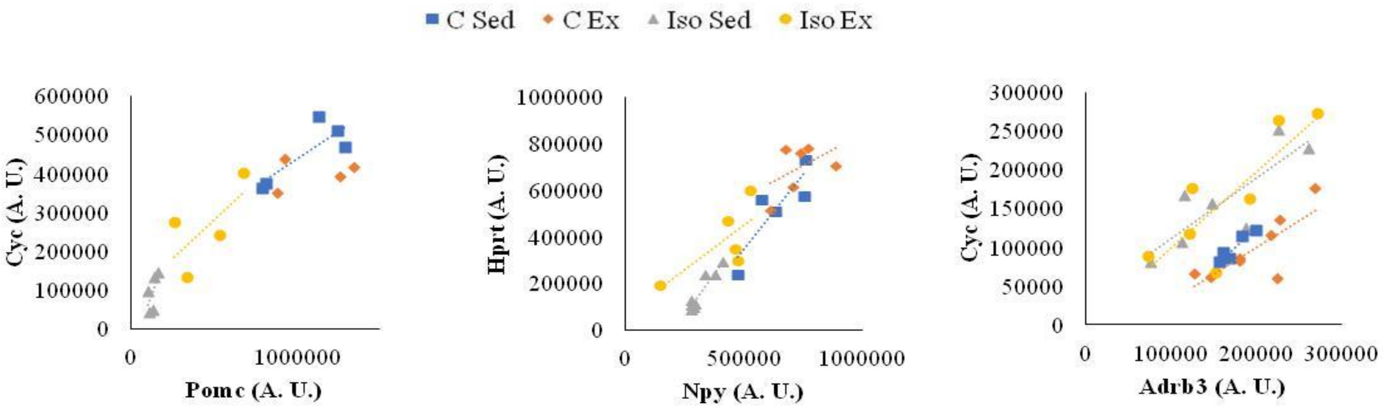

Supplementary Figure 2. *Oligonucleotides and RT PCR conditions of experimental genes*. Reference or gene accession number of sequences used as template, product size, PCR templates, annealing temperature, optimal cycles and cDNA. Graphs represent the correlations between a cDNA gene of experimental gene and housekeeping gene of one representative experiment.

- de Rijke CE, Hillebrand JJG, Verhagen LAW, Roeling TAP, Adan RAH. Hypothalamic neuropeptide expression following chronic food restriction in sedentary and wheel-running rats. *J Mol Endocrinol* (2005) **35**:381-390. doi: 10.1677/jme.1.01808.
- Leriche M, Cote-Vélez A, Méndez M. Presence of pro-opiomelanocortin mRNA in the rat medial prefrontal cortex, nucleus accumben and ventral tegmental area: Studies by RT-PCR and in situ hybridization techniques. *Neuropeptides* (2007) **41**:421-431. doi: 10.1016/j.npep.2007.08.004.
