# Supplementary material for: Voluntary Exercise-Induced Activation of Thyroid Axis and Reduction of White Fat Depots Is Attenuated by Chronic Stress in a Sex Dimorphic Pattern in Adult Rats
Source: Front Endocrinol (Lausanne). 2019 Jun 26;10:418. doi: 10.3389/fendo.2019.00418 (PMC6607407; doi:10.3389/fendo.2019.00418)
Supplement: Supplementary file 3 [file Image_3.pdf]

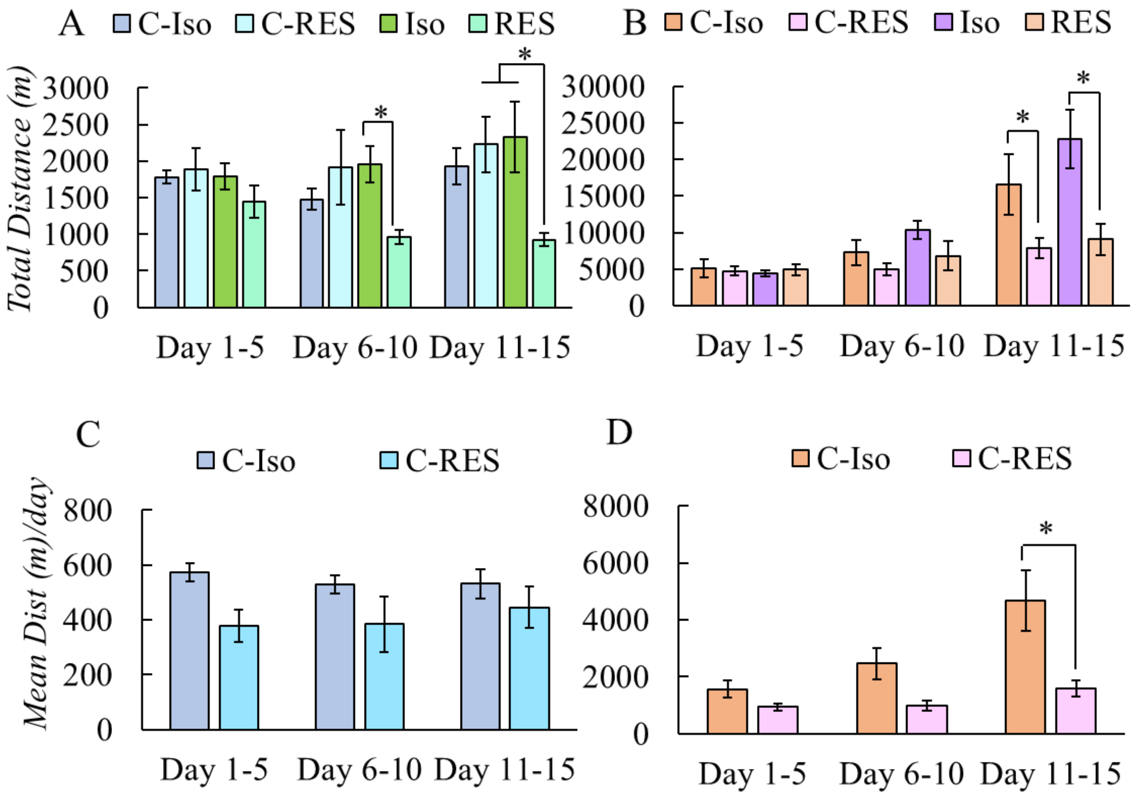

Supplementary Figure 3. *Comparison between physical activity of male and female of both experiments*. A, B: Physical activity of Iso, Res and respective controls rats. Results expressed in total revolutions at periods stated in the abscissa. C, D: Physical activity of control rats of both experiments. Results expressed in mean revolutions per day at periods stated in the abscissa. Significant followed by post hoc: * $P<0.05$.
