# Supplementary material for: Voluntary Exercise-Induced Activation of Thyroid Axis and Reduction of White Fat Depots Is Attenuated by Chronic Stress in a Sex Dimorphic Pattern in Adult Rats
Source: Front Endocrinol (Lausanne). 2019 Jun 26;10:418. doi: 10.3389/fendo.2019.00418 (PMC6607407; doi:10.3389/fendo.2019.00418)
Supplement: Supplementary file 4 [file Table_1.pdf]

Supplementary Table 1A. Results of statistical analyses on the effect of restraint before exercise period in both sexes.

	Sex		Restraint		Sex * Restraint	
	**F**	**P**	**F**	**P**	**F**	**P**
Food Intake (g/d)	$F_{1,54}$ (11742)	<0.001	$F_{2,54}$ (19.23)	<0.001	$F_{2,54}$ (19.6)	<0.001
Body weight gain (g)	$F_{1,54}$ (102.84)	<0.001	$F_{2,54}$ (4.36)	0.01	$F_{2,54}$ (2.94)	**0.061**
Relative Food Intake (g/d/kg)	$F_{1,54}$ (101.84)	<0.001	$F_{2,54}$ (6.44)	0.0031	$F_{2,54}$ (0.766)	0.469
Relative Body Weight gain (g/kg)	$F_{1,54}$ (34.797)	<0.001	$F_{2,54}$ (15.083)	<0.001	$F_{2,54}$ (16.53)	<0.001
Food Efficiency (g/100 g food)	$F_{1,54}$ (51.86)	<0.001	$F_{2,54}$ (9.82)	0.0002	$F_{2,54}$ (15.47)	<0.001

Supplementary Table 1B. Results of statistical analyses on the effect of housing before exercise period in both sexes.

	Sex		Housing		Sex * Housing	
	**F**	**P**	**F**	**P**	**F**	**P**
Food Intake PND 30-63 (g/day)	$F_{1,60}$ (265.82)	<0.001	$F_{1,60}$ (0.036)	0.850	$F_{1,60}$ (0.555)	0.459
Body Weight gain PND 30-63 (g)	$F_{1,60}$ (111.29)	<0.001	$F_{1,60}$ (0.720)	0.400	$F_{1,60}$ (0.056)	0.813
Relative Food Intake PND 30-63 (g/d/kg)	$F_{1,60}$ (835.83)	<0.001	$F_{1,60}$ (2.522)	0.118	$F_{1,60}$ (1.988)	0.164
Relative Body Weight gain PND 30-63 (g/kg)	$F_{1,60}$ (522.70)	<0.001	$F_{1,60}$ (0.767)	0.385	$F_{1,60}$ (0.406)	0.526
Food Efficiency PND 30-63 (g/100 g food)	$F_{1,60}$ (497.02)	<0.001	$F_{1,60}$ (2.299)	0.135	$F_{1,60}$ (0.665)	0.418
