# Supplementary material for: Voluntary Exercise-Induced Activation of Thyroid Axis and Reduction of White Fat Depots Is Attenuated by Chronic Stress in a Sex Dimorphic Pattern in Adult Rats
Source: Front Endocrinol (Lausanne). 2019 Jun 26;10:418. doi: 10.3389/fendo.2019.00418 (PMC6607407; doi:10.3389/fendo.2019.00418)
Supplement: Supplementary file 5 [file Table_2.pdf]

Supplementary Table 2A. Results of statistical analyses on the effect of restraint and physical activity in both sexes.

	Sex		Restraint		Activity		Sex*Restraint		Sex*Activity		Restraint*Activity		Sex*Restraint*Activity	
	**F**	**P**	**F**	**P**	**F**	**P**	**F**	**P**	**F**	**P**	**F**	**P**	**F**	**P**
Food Intake (g/d)	$F_{1,38}$ (15.08)	0.0004	$F_{1,38}$ (3.73)	**0.06**	$F_{1,38}$ (0.066)	0.798	$F_{1,38}$ (1.427)	0.239	$F_{1,38}$ (0.170)	0.682	$F_{1,38}$ (0.001)	0.972	$F_{1,38}$ (0.001)	0.977
Body weight gain (g)	$F_{1,38}$ (17.52)	0.0002	$F_{1,38}$ (2.8)	0.102	$F_{1,38}$ (0.748)	0.392	$F_{1,38}$ (0.054)	0.817	$F_{1,38}$ (1.42)	0.24	$F_{1,38}$ (0.204)	0.654	$F_{1,38}$ (0.026)	0.871
Relative Food Intake (g/d/kg)	$F_{1,38}$ (19.3)	<0.0001	$F_{1,38}$ (2.563)	0.118	$F_{1,38}$ (2.6E-4)	0.98	$F_{1,38}$ (0.032)	0.858	$F_{1,38}$ (0.001)	0.981	$F_{1,38}$ (0.069)	0.794	$F_{1,38}$ (0.335)	0.566
Relative Body Weight gain (g/kg)	$F_{1,38}$ (19.81)	<0.0001	$F_{1,38}$ (1.172)	0.285	$F_{1,38}$ (0.388)	0.53	$F_{1,38}$ (1.675)	0.203	$F_{1,38}$ (0.821)	0.37	$F_{1,38}$ (1.675)	0.203	$F_{1,38}$ (0.763)	0.388
Food Efficiency (g/100 g food)	$F_{1,38}$ (16.28)	0.0003	$F_{1,38}$ (0.831)	0.367	$F_{1,38}$ (0.266)	0.608	$F_{1,38}$ (2.769)	0.104	$F_{1,38}$ (1.257)	0.269	$F_{1,38}$ (1.79)	0.189	$F_{1,38}$ (2.84)	0.09
WAT gon (%)	$F_{1,36}$ (4.17)	0.04	$F_{1,36}$ (0.055)	0.8161	$F_{1,36}$ (7.41)	0.009	$F_{1,36}$ (2.38)	0.131	$F_{1,36}$ (0.095)	0.759	$F_{1,36}$ (2.38)	0.131	$F_{1,36}$ (0.119)	0.759
WAT retro (%)	$F_{1,36}$ (0.204)	0.65	$F_{1,36}$ (0.001)	0.927	$F_{1,36}$ (10.06)	0.003	$F_{1,36}$ (0.209)	0.65	$F_{1,36}$ (0.059)	0.81	$F_{1,36}$ (10.47)	0.0026	$F_{1,36}$ (0.444)	0.509
WAT inter (%)	$F_{1,36}$ (0.085)	0.77	$F_{1,36}$ (1.267)	0.267	$F_{1,36}$ (11.21)	0.0019	$F_{1,36}$ (0.062)	0.08	$F_{1,36}$ (0.154)	0.696	$F_{1,36}$ (7.686)	0.008	$F_{1,36}$ (0.873)	0.356
PVN: *Trh* mRNA (%)	$F_{1,35}$ (4.126)	0.04	$F_{1,35}$ (0.631)	0.432	$F_{1,35}$ (27.07)	<0.0001	$F_{1,35}$ (2.439)	0.127	$F_{1,35}$ (5.36)	0.026	$F_{1,35}$ (5.638)	0.0232	$F_{1,35}$ (1.822)	0.185
TSH (%)	$F_{1,34}$ (11.2)	0.002	$F_{1,34}$ (0.099)	0.754	$F_{1,34}$ (0.401)	0.53	$F_{1,34}$ (0.502)	0.483	$F_{1,34}$ (9.48)	0.004	$F_{1,34}$ (1.069)	0.308	$F_{1,34}$ (0.954)	0.335
T3 (%)	$F_{1,35}$ (4.408)	0.0431	$F_{1,35}$ (10.049)	0.003	$F_{1,35}$ (0.192)	0.66	$F_{1,35}$ (6.454)	0.015	$F_{1,35}$ (0.016)	0.9	$F_{1,35}$ (0.026)	0.872	$F_{1,35}$ (0.101)	0.752
T4 (%)	$F_{1,33}$ (2.57)	0.118	$F_{1,33}$ (1.709)	0.2	$F_{1,33}$ (2.87)	0.099	$F_{1,33}$ (3.937)	0.05	$F_{1,33}$ (2.981)	0.093	$F_{1,33}$ (1.749)	0.195	$F_{1,33}$ (3.467)	**0.0715**

Supplementary Table 2B. Results of statistical analyses on the effect of housing and physical activity in both sexes.

	Sex		Housing		Activity		Sex*Housing		Sex*Activity		Housing *Activity		Sex*Housing*Activity	
	**F**	**P**	**F**	**P**	**F**	**P**	**F**	**P**	**F**	**P**	**F**	**P**	**F**	**P**
Food Intake PND 63-79	$F_{1,50}$ (641.58)	<0.001	$F_{1,50}$ (12.367)	<0.001	$F_{1,50}$ (0.247)	0.603	$F_{1,50}$ (0.040)	0.842	$F_{1,50}$ (0.427)	0.516	$F_{1,50}$ (0.147)	0.703	$F_{1,50}$ (0.00001)	0.997
Body Weight gain PND 63-79	$F_{1,43}$ (33.826)	<0.001	$F_{1,43}$ (21.045)	<0.001	$F_{1,43}$ (2.459)	0.124	$F_{1,43}$ (4.457)	0.041	$F_{1,43}$ (0.111)	0.741	$F_{1,43}$ (0.013)	0.910	$F_{1,43}$ (0.779)	0.382
Relative Food intake PND 63-79 (g/d/kg)	$F_{1,50}$ (40.544)	<0.001	$F_{1,50}$ (4.848)	0.035	$F_{1,50}$ (0.001)	0.972	$F_{1,50}$ (1.037)	0.313	$F_{1,50}$ (0162)	0.689	$F_{1,50}$ (0.778)	0.382	$F_{1,50}$ (0.426)	0.517
Relative Body Weight gain PND 63-79 (g/kg)	$F_{1,49}$ (1.046)	0.312	$F_{1,49}$ (15.529)	<0.001	$F_{1,49}$ (3.035)	0.088	$F_{1,49}$ (1.405)	0.242	$F_{1,49}$ (0.019)	0.891	$F_{1,49}$ (0.141	0.709	$F_{1,49}$ (0.317)	0.576
Food Efficiency PND 63-79 (g/100 g food)	$F_{1,49}$ (14.360)	<0.001	$F_{1,49}$ (17.216)	<0.001	$F_{1,49}$ (3.410)	0.071	$F_{1,49}$ (2.890)	0.095	$F_{1,49}$ (0.208)	0.651	$F_{1,49}$ (0.368)	0.547	$F_{1,49}$ (0.222)	0.639
WAT gon (g/g BW)	$F_{1,48}$ (33.945)	<0.001	$F_{1,48}$ (9.246)	0.004	$F_{1,48}$ (2.274)	0.138	$F_{1,48}$ (0.586)	0.448	$F_{1,48}$ (2.084)	0.155	$F_{1,48}$ (0.965)	0.331	$F_{1,48}$ (0.974)	0.329
WAT retro (g/g BW)	$F_{1,50}$ (15.978)	<0.001	$F_{1,50}$ (6.539)	0.014	$F_{1,50}$ (0.914)	0.344	$F_{1,50}$ (0.659)	0.421	$F_{1,50}$ (0.570)	0.454	$F_{1,50}$ (0.468)	0.497	$F_{1,50}$ (0.192)	0.664
WAT inter (g/g BW)	$F_{1,50}$ (6.477)	0.014	$F_{1,50}$ (1.655)	0.204	$F_{1,50}$ (4.719)	0.035	$F_{1,50}$ (0.512)	0.477	$F_{1,50}$ (1.149)	0.289	$F_{1,50}$ (0.147)	0.703	$F_{1,50}$ (0.098)	0.756
Corticosterone (ng/ml)	$F_{1,44}$ (20.725)	<0.001	$F_{1,44}$ (7.551)	0.009	$F_{1,44}$ (1.942)	0.170	$F_{1,44}$ (3.825)	0.057	$F_{1,44}$ (0.384)	0.539	$F_{1,44}$ (1.122)	0.295	$F_{1,44}$ (0.925)	0.341
TSH (ng/ml)	$F_{1,53}$ (11.919)	0.001	$F_{1,53}$ (1.003)	0.321	$F_{1,53}$ (1.186)	0.281	$F_{1,53}$ (0.790)	0.378	$F_{1,53}$ (11.744)	0.001	$F_{1,53}$ (3.885)	0.054	$F_{1,53}$ (0.425)	0.517
T3 (ng/ml)	$F_{1,55}$ (310.22)	<0.001	$F_{1,55}$ (4.626)	0.036	$F_{1,55}$ (1.568)	0.216	$F_{1,55}$ (4.685)	0.035	$F_{1,55}$ (0.096)	0.758	$F_{1,55}$ (2.773)	0.102	$F_{1,55}$ (3.156)	0.081
T4 (ng/ml)	$F_{1,54}$ (32.260)	<0.001	$F_{1,54}$ (0.146)	0.703	$F_{1,54}$ (0.022)	0.882	$F_{1,54}$ (12.005)	0.001	$F_{1,54}$ (0.443)	0.508	$F_{1,54}$ (0.205)	0.652	$F_{1,54}$ (0.667)	0.418
T4:T3 ratio	$F_{1,54}$ (63.625)	<0.001	$F_{1,54}$ (0.036)	0.851	$F_{1,54}$ (1.475)	0.320	$F_{1,54}$ (11.659)	0.001	$F_{1,54}$ (1.432)	0.237	$F_{1,54}$ (0.014)	0.908	$F_{1,54}$ (2.278)	0.137

WAT gonadal (%)	$F_{1,46}$ (6.333)	0.015	$F_{1,46}$ (9.435)	0.004	$F_{1,46}$ (2.287)	0.137	$F_{1,46}$ (0.006)	0.937	$F_{1,46}$ (2.128)	0.151	$F_{1,46}$ (0.949)	0.335	$F_{1,46}$ (0.957)	0.333
WAT retro (%)	$F_{1,45}$ (3.929)	0.054	$F_{1,45}$ (5.291)	0.026	$F_{1,45}$ (1.421)	0.239	$F_{1,45}$ (1.060)	0.309	$F_{1,45}$ (0.987)	0.326	$F_{1,45}$ (0.651)	0.424	$F_{1,45}$ (0.091)	0.764
WAT inter (%)	$F_{1,48}$ (7.579)	0.008	$F_{1,48}$ (6.621)	0.112	$F_{1,48}$ (4.173)	0.047	$F_{1,48}$ (1.654)	0.205	$F_{1,48}$ (0.651)	0.437	$F_{1,48}$ (0.266)	0.609	$F_{1,48}$ (0.041)	0.841
PVN: *Crh* mRNA (%)	$F_{1,39}$ (17.034)	<0.001	$F_{1,39}$ (3.004)	0.091	$F_{1,39}$ (0.530)	0.471	$F_{1,39}$ (18.919)	<0.001	$F_{1,39}$ (0.161)	0.691	$F_{1,39}$ (0.183)	0.671	$F_{1,39}$ (0.388)	0.537
PVN: *Gr* mRNA (%)	$F_{1,43}$ (0.457)	0.503	$F_{1,43}$ (0.293)	0.591	$F_{1,43}$ (2.303)	0.136	$F_{1,43}$ (5.759)	0.021	$F_{1,43}$ (1.856)	0.180	$F_{1,43}$ (3.381)	0.073	$F_{1,43}$ (0.131)	0.720
Cort (%)	$F_{1,43}$ (4.758)	0.035	$F_{1,43}$ (7.733)	0.008	$F_{1,43}$ (3.899)	0.055	$F_{1,43}$ (0.0006)	0.980	$F_{1,43}$ (0.633)	0.431	$F_{1,43}$ (2.156)	0.149	$F_{1,43}$ (1.992)	0.165
MBH: *Pomc* mRNA (%)	$F_{1,28}$ (4.876)	0.036	$F_{1,28}$ (18.240)	<0.001	$F_{1,28}$ (15.031)	<0.001	$F_{1,28}$ (0.0004)	0.983	$F_{1,28}$ (12.028)	0.002	$F_{1,28}$ (1.461)	0.237	$F_{1,28}$ (1.534)	0.226
MBH: *Npy* mRNA (%)	$F_{1,45}$ (0.626)	0.433	$F_{1,45}$ (8.649)	0.005	$F_{1,45}$ (2.136)	0.151	$F_{1,45}$ (16.537)	<0.001	$F_{1,45}$ (17.292)	<0.001	$F_{1,45}$ (25.590)	<0.001	$F_{1,45}$ (0.799)	0.382
MBH: *Dio2* mRNA (%)	$F_{1,40}$ (1.795)	0.188	$F_{1,40}$ (0.001)	0.972	$F_{1,40}$ (0.065)	0.800	$F_{1,40}$ (0.165)	0.687	$F_{1,40}$ (10.612)	0.002	$F_{1,40}$ (0.054)	0.817	$F_{1,40}$ (17.568)	<0.001
MBH: *Trhde* mRNA (%)	$F_{1,24}$ (0.819)	0.374	$F_{1,24}$ (21.963)	<0.001	$F_{1,24}$ (8.594)	0.007	$F_{1,24}$ (10.072)	0.004	$F_{1,24}$ (4.697)	0.040	$F_{1,24}$ (0.473)	0.498	$F_{1,24}$ (3.654)	0.068
PVN: *Trh* mRNA (%)	$F_{1,43}$ (4.853)	0.033	$F_{1,43}$ (0.245)	0.623	$F_{1,43}$ (1.880)	0.177	$F_{1,43}$ (0.066)	0.798	$F_{1,43}$ (3.203)	0.081	$F_{1,43}$ (5.648)	0.022	$F_{1,43}$ (0.024)	0.877
TSH (%)	$F_{1,52}$ (3.350)	0.073	$F_{1,52}$ (1.207)	0.277	$F_{1,52}$ (0.040)	0.842	$F_{1,52}$ (1.946)	0.169	$F_{1,52}$ (10.789)	0.002	$F_{1,52}$ (3.240)	0.078	$F_{1,52}$ (0.004)	0.953
T3 (%)	$F_{1,55}$ (0.538)	0.538	$F_{1,55}$ (3.756)	0.058	$F_{1,55}$ (3.047)	0.086	$F_{1,55}$ (4.872)	0.034	$F_{1,55}$ (0.110)	0.741	$F_{1,55}$ (2.059)	0.157	$F_{1,55}$ (2.823)	0.099
T4 (%)	$F_{1,54}$ (10.783)	0.002	$F_{1,54}$ (0.026)	0.873	$F_{1,54}$ (0.037)	0.847	$F_{1,54}$ (11.938)	0.001	$F_{1,54}$ (0.460)	0.500	$F_{1,54}$ (0.258)	0.614	$F_{1,54}$ (0.722)	0.399
BAT: *Adrb3* mRNA (%)	$F_{1,27}$ (0.905)	0.350	$F_{1,27}$ (1.410)	0.245	$F_{1,27}$ (14.664)	<0.001	$F_{1,27}$ (5.918)	0.022	$F_{1,27}$ (2.728)	0.110	$F_{1,27}$ (0.042)	0.840	$F_{1,27}$ (0.029)	0.866
BAT: *Dio2* mRNA (%)	$F_{1,29}$ (8.747)	0.006	$F_{1,29}$ (1.308)	0.262	$F_{1,29}$ (3.913)	0.057	$F_{1,29}$ (0.665)	0.421	$F_{1,29}$ (9.867)	0.004	$F_{1,29}$ (1.035)	0.317	$F_{1,29}$ (0.999)	0.326
BAT: *Ucp1* mRNA (%)	$F_{1,31}$ (3.353)	0.077	$F_{1,31}$ (1.446)	0.238	$F_{1,31}$ (19.155)	<0.001	$F_{1,31}$ (13.447)	<0.001	$F_{1,31}$ (0.329)	0.570	$F_{1,31}$ (4.736)	0.037	$F_{1,31}$ (1.593)	0.216
