# Supplementary material for: Voluntary Exercise-Induced Activation of Thyroid Axis and Reduction of White Fat Depots Is Attenuated by Chronic Stress in a Sex Dimorphic Pattern in Adult Rats
Source: Front Endocrinol (Lausanne). 2019 Jun 26;10:418. doi: 10.3389/fendo.2019.00418 (PMC6607407; doi:10.3389/fendo.2019.00418)
Supplement: Supplementary file 6 [file Table_3.pdf]

Supplementary Table 3A. Relative food intake (RFI), relative body weight gain (RBWg) and food efficiency (FE) during restraint stress period of naïve (N), pair-fed control (C) and restraint (Res) male and female rats.

**Males**	N	C	Res
RFI (g/d/kg)	63.2 ± 1.7	63.9 ± 1.7	59.7 ± 1.4
RBWg (g/kg)	69.9 ± 4.0	63.7 ± 7.5	57.09 ± 4.9
FE (g/100 g food)	7.8 ± 0.6	7.1 ± 0.8	6.0 ± 0.6
**Females**			
RFI (g/d/kg)	79.0 ± 2.4^{A}	75.8 ± 1.3^{A}	71.9 ± 1.03*°^{A}
RBWg (g/kg)	52.2 ± 13.0^{A}	-16.10 ± 9.9°^{A}	47.85 ± 5.6*^{A}
FE (g/100 g food)	6.0 ± 2.6^{A}	-0-53± 0.46°^{A}	1.99 ± 0.54*^{A}

Male and female Wistar adult rats were restrained (R) or kept in single housing for 30 min/day for 14 consecutive days in two independent rooms, food intake of restrained group was pair-fed to control group (C). Results are expressed in mean ± SEM. Significant ANOVAs (Supplentary Table 1A) followed by post hoc: * *P<0.05* vs. C group; ^{A} *P<0.05* vs. Sex; ° *P<0.05* vs N.

Supplementary Table 3B. Relative food intake (RFI), relative body weight gain (RBWg) and food efficiency (FE) during adolescence-adulthood period of group-housed (C) and isolated (Iso) male and female rats.

**Males**	C	Iso
RFI (g/d/kg)	80.82 ± 1.47	82.37 ± 1.00
RBWg (g/kg)	678.71 ± 6.32	667.98 ± 5.49
FE (g/100 g food)	30.32 ± 0.53	29.20 ± 0.50
**Females**		
RFI (g/d/kg)	96.20 ± 2.06^{A}	97.08 ± 0.87^{A}
RBWg (g/kg)	512.09 ± 8.86^{A}	510.39 ± 7.25^{A}
FE (g/100 g food)	19.21 ± 0.55^{A}	18.87 ± 0.30^{A}

Male and female Wistar rats were housed isolated (Iso) or in 2/cage (Controls, C) from PND30 to PND63. Results are expressed in mean ± SEM. Significant ANOVAs (Supplementary Table 1B) followed by post hoc: ^{A} *P<0.001* vs. Sex.
