# Supplementary material for: Voluntary Exercise-Induced Activation of Thyroid Axis and Reduction of White Fat Depots Is Attenuated by Chronic Stress in a Sex Dimorphic Pattern in Adult Rats
Source: Front Endocrinol (Lausanne). 2019 Jun 26;10:418. doi: 10.3389/fendo.2019.00418 (PMC6607407; doi:10.3389/fendo.2019.00418)
Supplement: Supplementary file 7 [file Table_4.pdf]

Supplementary Table 4A. Relative food intake (RFI), relative body weight gain (RBWg) and food efficiency (FE) after exercise period. Effect of restraint.

**Males**	N	C pf	C ex	Res pf	Res ex
RFI (g/d/kg)	56.77 ± 1.5	43.1 ± 1.8°	44.1 ± 3.14°	49.3 ± 2.6	48.4 ± 3.2
RBWg (g/kg)	45.3 ± 5.5	-15.6 ± 9.7°	-32.07 ± 7.5°	5.66 ± 10.8	-23.2 ± 24.0°
FE (g/100 g food)	7.1 ± 0.78	2.8 ± 2.7	-3.7 ± 1.46°	-2.5 ± 1.8*°	-0.6 ± 1.49°
**Females**					
RFI (g/d/kg)	82.95 ± 2.3^A	58.31 ± 4.8°^{A}	55.81 ± 3.6°	60.00 ± 4.7°^{A}	62.00 ± 4.7°^{A}
RBWg (g/kg)	52.00 ± 20.00	22.97 ± 14.00^A	41.49 ± 23.48	72.14 ± 20.78°^{A}	44.21 ± 22.98^A
FE (g/100 g food)	4.66 ± 1.99	2.05 ± 1.8	3.36 ± 0.87^A	6.35 ± 1.5^A	6.7 ± 1.44^A

Restrained or control rats were either exposed to a running wheel (C ex and Res ex) or left individually in a cage (C pf and Res pf) overnight for 14 days. Food intake of exercised animals was pair-fed to control groups. Significant ANOVAs (Supplementary Table 2A) followed by post hoc: ^{A} *P<0.05* vs. Sex; ° *P<0.05* vs. N.

Supplementary Table 4B. Relative food intake (RFI), relative body weight gain (RBWg) and food efficiency (FE) after exercise period. Effect of social isolation.

**Males**	C-Sed	C-Ex	Iso-Sed	Iso-Ex
RFI (g/d/kg)	63.31 ± 1.78	66.09 ± 2.09	70.26 ± 1.98*	68.56 ± 0.91*
RBWg (g/kg)	43.01 ± 4.23	38.22 ± 9.19	79.26 ± 7.06*	65.15 ± 5.26*
FE (g/100 g food)	3.20 ± 1.04	3.94 ± 1.22	9.19 ± 0.67*	6.60 ± 1.33*
**Females**				
RFI (g/d/kg)	75.65 ± 2.20^A	75.35 ± 3.27^A	77.72 ± 1.72*^{A}	76.74 ± 2.21*^{A}
RBWg (g/kg)	48.84 ± 14.05	31.72 ± 16.06	61.07 ± 6.90*	56.59 ± 8.6*
FE (g/100 g food)	4.26 ± 1.16^A	2.68 ± 1.44^A	5.14 ± 0.52*^{A}	4.94 ± 0.79*^{A}

At PND 63, group-housed (C) and isolated (Iso) rats were separated in two groups, one was left undisturbed (Sedentary, Sed) and the other was exposed to a running wheel in alternated days during the dark period. Sed group received the amount of food that Ex group. Results are expressed in mean ± SEM. Significant ANOVAs (Supplementary table 2B) followed by post hoc: * *P<0.05* vs. C group; $^{\&}$ *P<0.05* vs. Sed group; ^{A} *P<0.001* vs. Sex of same group.
