# Supplementary material for: Voluntary Exercise-Induced Activation of Thyroid Axis and Reduction of White Fat Depots Is Attenuated by Chronic Stress in a Sex Dimorphic Pattern in Adult Rats
Source: Front Endocrinol (Lausanne). 2019 Jun 26;10:418. doi: 10.3389/fendo.2019.00418 (PMC6607407; doi:10.3389/fendo.2019.00418)
Supplement: Supplementary file 8 [file Table_5.pdf]

**Supplementary Table 5.** Absolute values of relative fat mass and hormone concentration after exercise. Effect of social isolation.

| **Males** | C-Sed | C-Ex | Iso-Sed | Iso-Ex |
|---|---|---|---|---|
| WAT gonadal (g/g BW) | 0.0119 ± 0.0005 | 0.0119 ± 0.0007 | 0.0147 ± 0.010* | 0.0146 ± 0.009* |
| WAT retro (g/g BW) | 0.0103 ± 0.0007 | 0.0092 ± 0.0008 | 0.0123 ± 0.0006* | 0.0132 ± 0.0018* |
| WAT inter (g/g BW) | 0.0023 ± 0.0002 | 0.0020 ± 0.0002 | 0.0028 ± 0.0005 | 0.0025 ± 0.0003 |
| Cort (ng/ml) | 74.06 ± 21.37 | 148.12 ± 26.72 | 106.62 ± 31.14* | 73.59 ± 14.43* |
| TSH (ng/ml) | 2.06 ± 0.24 | 1.80 ± 0.26[&] | 2.40 ± 0.26 | 1.44 ±0.15[&] |
| T3 (ng/ml) | 1.84 ± 0.14 | 2.15 ± 0.15 | 1.79 ± 0.07 | 1.67 ± 0.11* |
| T4 (mg/dl) | 12.20 ± 0.80 | 12.29 ± 0.45[&] | 14.37 ± 0.85* | 14.92 ± 1.39*[&] |
| T4:T3 ratio | 6.851 ± 0.676 | 5.907 ± 0.648 | 8.076 ± 0.524* | 8.996 ± 0.719* |
| **Females** | | | | |
| WAT gonadal (g/g BW) | 0.0100 ± 0.0007[A] | 0.0065 ± 0.0006[A] | 0.0102 ± 0.0014[A] | 0.0095 ± 0.0015*[A] |
| WAT retro (g/g BW) | 0.0078 ± 0.0008[A] | 0.0060 ± 0.0007[A] | 0.0091 ± 0.0019*[A] | 0.0078 ± 0.0017*[A] |
| WAT inter (g/g BW) | 0.0035 ± 0.0004[A] | 0.0025 ± 0.0002[&] | 0.0034 ± 0.0004[A] | 0.0028 ± 0.0005[&] |
| Cort (ng/ml) | 255.56 ± 22.00[A] | 311.47 ± 72.61[A] | 133.52 ± 13.14*[A] | 184.28 ± 20.59*[A] |
| TSH (ng/ml) | 1.34 ± 0.14 | 1.83 ± 0.13[A] | 1.26 ± 0.14* | 1.40 ±0.10*[A] |
| T3 (ng/ml) | 0.76 ± 0.02[A] | 0.81 ± 0.04[&A] | 0.75 ± 0.02[A] | 0.82 ± 0.02[&A] |
| T4 (mg/dl) | 10.72 ± 1.04[A] | 11.01 ± 0.61[A] | 9.59 ± 0.59[A] | 8.29 ± 0.89*[A] |
| T4:T3 ratio | 14.259 ± 1.520[A] | 13.734 ± 0.828[A] | 12.936 ± 0.978*[A] | 10.237 ± 1.129*[A] |

At PND 63, group-housed (C) and isolated (Iso) rats were separated in two groups, one was left undisturbed (Sedentary, Sed) and the other was exposed to a running wheel in alternated days during the dark period. Sed group received the amount of food that Ex group. Results are expressed in mean ± SEM. Significant ANOVAs (Supplementary Table 2B) followed by post hoc: * *P<0.05* vs. C group; [&] *P<0.05* vs. Sed group; [A] *P<0.001* vs. Sex of same group.
